# The Effect of High Pressure Processing on Polyphenol Oxidase Activity, Phytochemicals and Proximate Composition of Irish Potato Cultivars †

**DOI:** 10.3390/foods8100517

**Published:** 2019-10-19

**Authors:** Konstantina Tsikrika, Nora O’Brien, Dilip K. Rai

**Affiliations:** 1 Department of Food Biosciences, Teagasc Food Research Centre, Ashtown, D15 KN3K Dublin, Ireland; Konstantina.tsikrika@teagasc.ie; 2 School of Food & Nutritional Sciences, University College Cork, College Road, T12 K8AF Cork, Ireland; nob@ucc.ie

**Keywords:** high pressure processing, potatoes, polyphenol oxidase, polyphenols, glycoalkaloids

## Abstract

Polyphenol oxidase (PPO) activity, proximate composition, and phytochemicals were determined in four common Irish potato cultivars following a high pressure processing (HPP) at 600 MPa for 3 min. PPO activity was significantly (*p* < 0.05) lower in all HPP treated samples, while the overall proximate composition was not affected. The total phenolic content was significantly higher in the HPP treated samples. Chlorogenic acid levels significantly decreased with simultaneous increase of caffeic acid and *p*-coumaric acid levels upon HPP treatment. No significant changes were observed in rutin and ferulic acid levels, although their levels varied, depending on the potato cultivars, while the levels of cytotoxic glycoalkaloids (α-solanine and α-chaconine) remained unaltered.

## 1. Introduction

Potatoes (*Solanum tuberosum* L.) are considered a staple nutritional diet, owing to high quality proteins, low in fat, and rich in carbohydrates and dietary fibre that promote human health [1]. Potatoes are also excellent sources of dietary antioxidants, such as polyphenols, ascorbic acid, carotenoids, tocopherols, α-lipoic acid, and selenium [2], which are involved in immunomodulatory reactions that are related with the prevention of various diseases, such as cancer, cardiovascular, and neurodegenerative diseases [3]. Potatoes also contain toxic glycoalkaloids, namely α-chaconine and α-solanine, and when consumed in excess (>1 mg glycoalkaloids per kg body weight) can cause from mild to severe symptoms [4]. 

Polyphenol oxidase (PPO) activity is involved in the browning reaction in minimally processed potatoes. Phenolic compounds that are present in potatoes or other fruits and vegetables are oxidized by PPO to quinones, which are then polymerized to melanin pigments, resulting in the development of undesirable colours and texture as well as losses of flavour and nutrients [5]. High pressure processing (HPP) presents a viable alternative to conventional thermal processes regarding PPO inactivation and, at the same time enabling a more wholesome, fresh product with extended shelf-life [6]. 

Currently, HPP has been successfully employed to various fruit and vegetable purées and juices [7,8,9,10,11,12,13,14], fruit juice based beverages [15,16], smoothies [17], milk [18,19] cocoyam, Peruvian carrot and sweet potato [20] purple sweet potato nectar [21], peaches [22,23], and pumpkin [24]. In our previous work, we have shown that HPP had no significant (*p* > 0.05) impact on the antioxidant activity of potatoes [25]. However, the effect of HPP on PPO inactivation, proximate composition, and bioactive phytochemicals (polyphenols and glycoalkaloids) of potatoes has not been explored. Hence, the aim of this work is to examine the effect of HPP on these attributes of the potatoes of two coloured (Rooster and Kerr’s Pink) and two white (Gemson and Saxon) cultivars, which are commonly consumed in the island of Ireland. 

## 2. Materials and Methods

### 2.1. Samples

Freshly harvested potatoes (*Solanum tuberosum* L.) of Rooster cultivar were provided by Country Crest Ltd., Lusk, Co. Dublin. Saxon, Gemson and Kerr’s Pink potatoes were purchased from a local market in Dublin, Ireland. The potatoes were stored at 4 °C, vacuum packed in polyethylene/polyamide pouches before and after the HPP treatment and until further analysis.

### 2.2. Chemicals

Hydrochloric acid (HCl), sodium phosphate monobasic, sodium phosphate dibasic, catechol, Folin & Ciocalteu’s phenol reagent, gallic acid, methanol, acetonitrile, sodium carbonate, formic acid, petroleum ether, ethanol, sodium azide, amylase, protease, amyloglucosidase, diatomaceous earth (celite 545 AW), 2-(*N*-Morpholino)ethanesulfonic acid, tris(hydroxymethyl)aminomethane, acetone, and the phytochemical standards (quinic acid, chlorogenic acid, caffeic acid, rutin, *p*-coumaric acid, ferulic acid, α-solanine, α-chaconine) were purchased from Merck Ltd. (formerly Sigma Aldrich Ltd, Wicklow, Ireland). Leucine-enkelphine was procured from VWR International Ltd. (Blanchardstown, Dublin, Ireland).

### 2.3. High Pressure Processing 

Potatoes of different cultivars were packaged in polyethylene/polyamide pouches and then vacuum sealed. HPP treatment was performed at 600 MPa (6000 bar) for 3 min. at 10. 6 °C (max. temperature reached) on commercial-scale high pressure press (Hiperbaric 55HT, Doral, FL, USA) housed at HPP Tolling (St. Margarets, Co. Dublin, Ireland). 

### 2.4. Potato PPO Extraction and Activity Assay

PPO extraction and activity assay were performed according to [26] with small modifications. Potato (20 g) was homogenized with cold phosphate buffer (40 mL; 0.1 M; pH 6.5; 4 °C) for 3 min. Afterwards, the homogenate was centrifuged at 10,000× *g* at 4 °C for 25 min. and the supernatant, constituting the crude extract, was used to assay PPO activity.

PPO activity reaction mixture contained phosphate buffer (1.5 mL; 0.1 M, pH 5.5), catechol (1 mL; 0.2 M) and crude PPO extract (0.5 mL). The absorbance was then measured at 410 nm for 2 min. by a spectrophotometer (UV-1700, Shimadzu, Ziangsu, China). The residual activity (RA) of PPO was calculated, as follows:

RA (%)= (A_t_/A_0_) ×100, (1)

where A_t_ and A_0_ are, respectively, PPO activity after and before the treatment.

### 2.5. Proximate Analysis

Proximate analysis was performed according to of the Association of Official Analytical Chemists (AOAC, 1990). Briefly, the potatoes were washed, cut, and oven dried at 103 °C for 4 h. The difference between the sample weight before and after the drying was used to calculate the moisture content. The dried samples were then milled while using a Robot Coupe and the milled samples were used for further analysis. The protein content was defined while using LECO FP628. The fat content was analysed by automated acid hydrolysis (ANKOM HCl Hydrolysis System, ANKOM Technology). The total dietary fibre was measured by ANKOM TDF Dietary Fiber Analyzer (ANKOM Technology). The ash content was determined by incineration in a carbolite furnace at 600 °C (AOAC No. 923.03). The available carbohydrate was calculated by difference. 

### 2.6. Extraction of Phytochemicals

Extraction procedure of phytochemicals was adapted from [27]. Freeze-dried potato flesh powder (~1 g) was mixed with 80% methanol (4 mL) containing 1% formic acid for overnight at 4 °C. The mixture was then sonicated at 30 °C for 30 min., and centrifuged at 10,000× *g* for 30 min. The residue was re-extracted with 80% methanol (2.5 mL) containing 1% formic acid, followed by centrifugation. The supernatants were combined and filtered through a 0.45 µm syringe filter.

### 2.7. Total Phenolic Content 

Total phenolic content (TPC) was colourimetrically assessed while using the Folin-Ciocalteu reagent (FCR) method [28]. The method was modified in order to be performed in a plate reader. Potato extract (100 µL) was mixed with FCR (100 µL), sodium carbonate (100 µL; 20% *w/v*) and methanol (100 µL; 80% *v/v*). Gallic acid was used as a standard and the absorbance was measured at 735 nm while using a plate reader (FLUOstar Omega Microplate Reader, BMG Labtech GmbH, Offenburg, Germany). The results were expressed as µg of gallic acid equivalent per g of dry weight of the extract (µg GAE/g dw).

### 2.8. Liquid Chromatography-Mass Spectrometry Analysis 

Quadrupole time-of-flight (Q-ToF) Premier mass spectrometer coupled to Alliance 2695 HPLC system (Waters Corporation, Milford, MA, USA) was used to profile various phytochemicals in the potato extracts following the procedure that was described by Hossain et al. (2010) [29]. Accurate mass measurements of the compounds and their fragment ions were determined while using a reference compound leucine–enkephalin. The separation of the analytes was performed on an Atlantis T3 C18 column (100 × 2.1 mm; 3 µm) while using solvent A (water 0.1% formic acid) and solvent B (0.1% in acetonitrile at a flow rate of 0.3 mL/min. at 40 °C. Electrospray ionisation (ESI) mass spectra were recorded on a negative ion mode for a mass range m/z 70–1000. Collision-induced dissociation (CID) was performed while using argon with collision energy ranging between 12–20 eV.

Ultra-high performance liquid chromatography coupled to tandem quadrupole mass spectrometer (UPLC-TQD, Waters Corp., Milford, MA, USA) was used to quantify the phytochemicals that were identified by the HPLC-Q-ToF analysis. Separation of the natural compounds in the potato extracts was achieved on a Waters Acquity UPLC HSS T3 column (100 × 2.1 mm, 1.8 µm) while using solvent A (water containing 0.1% formic acid) and solvent B (0.1% formic acid in acetonitrile at 0.5 mL/min. The UPLC-MS/MS conditions were as described previously for polyphenols [30] and glycoalkaloids [31]. Detection and quantification of the phenolic compounds in the UPLC-TQD were conducted in multiple reaction monitoring (MRM) mode by analysing 2–3 transitions per natural compound. The cone voltages and collision energies were optimised for MRM transition while using IntelliStart^TM^ software (Masslynx 4.1, Waters Corp., Milford, MA, USA). Analyses were carried out in triplicate extracts and target compounds were quantified using standard calibration curves of concentrations that ranged from 10 ng/mL–25 µg/mL. The results were expressed as µg compound per g of extract in dry weight (µg/g dw).

### 2.9. Statistical Analysis

Results are expressed as means triplicates replicated twice (*n* = 6) ± standard deviation (SD). Differences between means were analysed using independent samples *t*-test, while correlations between the variables were evaluated by Pearson correlation test. All of the statistical analyses were performed by SPSS Statistics 24 (IBM-Armonk, New York, NY, USA). The values were considered to be significantly different when *p* < 0.05. 

## 3. Results and Discussion

### 3.1. PPO Inactivation

HPP resulted in a significant (*p* < 0.05) decrease of PPO residual activity (RA) in all of the studied varieties. Specifically, PPO RA was found to be 41% in Saxon, 53% in Gemson, 49 in % Kerr’s Pink, and 31% in Rooster upon HPP treatment. Wang et al. (2012) [32] observed a much lower reduction of PPO activity of ~34.5% in a spinach-puree following HPP treatment also at 600 MPa, but for a longer duration (15 min.). High pressure–induced alterations of PPO structure involve partial or complete unfolding of the native structure, resulting in lower enzyme activity [33,34], which can explain our findings.

### 3.2. Proximate Analysis

Table 1 shows moisture, protein, fat, total dietary fibre, ash, and total carbohydrate content of potato samples before and after the HPP treatment. Moisture and protein contents were significantly decreased in all of the HPP treated samples when compared to those untreated potatoes. This can be attributed to the compression of the matrix during HPP treatment [35], leading to water and, consequently, protein loss. On the other hand, HPP did not have a statistically significant effect on the fat, total dietary fibre, ash, or total carbohydrate levels of the studied potato samples.

### 3.3. Effect of HPP on Total Phenolic Content

HPP significantly increased the total phenolic content (TPC) by approximately 21%, 24%, 23%, and 17% in Saxon, Gemson, Rooster, and Kerr’s Pink potatoes, respectively, with respect to those untreated (Table 2). However, changes in the antioxidant activities were non-significant between the HPP treated and untreated potatoes [25], which was also observed by Wang et al. 2012 in purple sweet potato nectars following HPP treatments. Significant increases in the TPC values following the HPP treatments have been observed in several fruits (or their products) and vegetables, including smoothies [17], pumpkins [24], pomegranate juices [10], fruit juice beverages [15], and strawberry puree [7]. The reported increase in the TPC can be attributed to an enhanced cell permeability by the disruption of the cell walls as well of the cell membrane hydrophobic bonds, leading to mass transfer, and the release of matrix-bound phenolic compounds [8,21]. 

### 3.4. Effect of HPP on Phytochemicals

A total of thirteen different phytochemicals have been detected in the potato extracts, with five of those being polyphenols and two glycoalkaloids (Supplementary Figure S1, Table S1). Their identification was achieved through accurate mass measurements of the compounds and their CID fragments, while using either authentic standards (quinic acid, chlorogenic acid, caffeic acid, rutin, *p*-coumaric acid, ferulic acid, α-solanine, α-chaconine) or information from existing literature. 

#### 3.4.1. Effect of HPP on Polyphenols

The levels of individual polyphenols in untreated and HPP potato samples were quantified by UPLC-TQD and are shown in Table 2, Figure S2). The most common phenolic acids, namely caffeic, *p*-coumaric, and ferulic acids, generally occur in foods as esters with quinic acid or sugar components of the food matrix [36]. Chlorogenic acid, an ester of caffeic acid with quinic acid, was the most abundant polyphenol in potatoes and it was found to range from 119 ± 14 µg/g dw to 140 ± 10 µg/g dw in the untreated potato samples, which is in accordance with existing literature [37]. HPP resulted in a significant (*p* < 0.05) decrease in chlorogenic acid of all potato varieties studies (89 ± 2 µg/g dw–120 ± 4 µg/g dw). Chlorogenic acid in potatoes has been previously found to be affected by various processing methods [38]. In parallel to chlorogenic acid’s decrease, a significant (*p* < 0.05) increase in caffeic acid was observed in all HPP treated potato samples (2.7 ± 0.1 µg/g–9.9 ± 0.1 µg/g), as opposed to the controls (0.1 ± 0.01 µg/g–1.2 ± 0.2 µg/g). This suggests that the free-form chlorogenic acid in potatoes is degraded to its constituent phenolic acid, i.e., caffeic acid. There was also a slight (~0.6–4%) but significant (*p* < 0.05) increase in the *p*-coumaric acid levels of the HPP treated potato samples, as compared to those untreated. Similarly, the levels of ferulic acid in Saxon and Kerr’s Pink were also slightly, but not significantly, increased in the post-HPP treated samples, suggesting that ferulic acid in these cultivars also occurs in bound form. On contrary, the decreased levels of ferulic acid in Gemson and Rooster in the HPP samples suggest that it occurs in free form. HPP can induce alterations to the tissue matrix, for instance, disruption of plant cell walls, followed by release of compounds into the extracellular environment [39], which might explain the observed increase of these hydroxycinnamic acids in the present study. Jeż et al. (2018) treated tomato purées from different commercial varieties with HPP at 450, 550, and 650 MPa for 5, 10, and 15 min. and observed remarkable losses in individual polyphenols, such as chlorogenic and isochlorogenic acid, caffeic acid, *p*-coumaric acid, and ferulic acid [40]. In the present study, the concentration of rutin in HPP treated potato samples did not change significantly (*p* > 0.05) as compared to the controls, although the levels varied between the different cultivars following post-HPP, indicating that rutin is present in Saxon and Kerr’s Pink in free-form, whereas it also occurs in bound form in Gemson and Rooster. Similar observations were made by Navarre et al. (2010) in the rutin levels of young potato tubers after different cooking methods, such as microwave, boiling, steaming, and baking [41]. 

#### 3.4.2. Effect of HPP on Glycoalkaloids

Steroidal alkaloids (glycoalkaloids) are nitrogenous secondary metabolites that are found in potatoes and other members of the Solanaceae family. The main glycoalkaloids in potatoes are α-chaconine and α-solanine, constituting from approximately 1 ± 0.2 µg/g–16.5 ± 1.5 µg/g and 1.5 ± 0.1 µg/g–18 ± 1.7 µg/g, respectively, which is in accordance with the literature [31]. However, HPP did not have a statistically significant effect on the glycoalkaloid content of the studied potato samples, i.e. within the same cultivar (Table 2).

## 4. Conclusions

High pressure processing (600 MPa, 3 min.) significantly decreased the PPO activity of the potato cultivars studied without significant effect on their proximate composition. Although the TPC was higher (*p* < 0.05) in the HPP treated samples, but there were no statistical differences in their antioxidant activities with respect to controls. Chlorogenic acid, the most abundant polyphenol associated with browning, significantly decreased with a simultaneous increase of smaller phenolic acids (caffeic acid and *p*-coumaric acid) in the HPP treated samples. Other minor polyphenols, namely rutin and ferulic acid levels, varied depending on the potato cultivars. Similarly, the levels of toxic glycoalkaloids α-solanine and α-chaconine did not change in the HPP treated samples, as opposed to the untreated samples. These findings portray that the HPP is a potential processing technique to reduce browning enzyme PPO with little impact on proximate composition, polyphenols, and glycoalkaloids in potatoes.

## Figures and Tables

**Table 1:** Proximate composition in percentages (%) of untreated (control) and treated with high pressure processing (HPP) potato cultivars.

Potato Cultivar	Sample Type	Moisture	Tot al Dietary Fibre	Protein	Fat	Ash	Tot al Carbohydrates
Saxon	control	76.07 ± 1.8 ^a^	32.7 ± 0.9 ^a^	1.86 ± 0.05 ^a^	0.36 ± 0.16 ^a^	2.18 ± 0.17 ^a^	19.23 ± 2.9 ^a^
	HPP-treated	74.74 ± 2.8 ^a^	31.2 ± 0.9 ^a^	1.27 ± 0.02 ^b^	0.23 ± 0.04 ^a^	1.90 ± 0.01 ^a^	21.95 ± 3.7 ^a^
Gemson	control	81.54 ± 0.4 ^b^	27.8 ± 0.1 ^b^	2.18 ± 0.07 ^c^	0.18 ± 0.08 ^a^	4.07 ± 0.009 ^b^	16.04 ± 0.4 ^b^
	HPP-treated	79.46 ± 1.4 ^c^	28.0 ± 2.1 ^b^	1.53 ± 0.09 ^d^	0.1 ± 0.03 ^a^	3.79 ± 0.28 ^b^	15.32 ± 1.6 ^b^
Rooster	control	72.48 ± 1.8 ^d^	13.5 ± 2.3 ^c^	2.29 ± 0.11 ^c^	0.16 ± 0.07 ^a^	3.60 ± 0.01 ^b^	21.47 ± 1.9 ^a^
	HPP-treated	72.18 ± 0.1 ^d^	15.3 ± 0.7 ^c^	1.91 ± 0.05 ^a^	0.21 ± 0.04 ^a^	3.43 ± 0.18 ^b^	23.06 ± 3.7 ^a^
Kerr’s Pink	control	79.40 ± 1.3 ^c^	29.6 ± 0.6 ^b^	2.07 ± 0.12 ^c^	0.15 ± 0.06 ^a^	3.62 ± 0.19 ^b^	20.48 ± 0.11 ^a^
	HPP-treated	73.77 ± 0.2 ^d^	28.5 ± 0.6 ^b^	1.48 ± 0.09 ^d^	0.13 ± 0.03 ^a^	3.53 ± 0.21 ^b^	19.55 ± 1.5 ^a^

Values are mean ± standard deviation. Mean values in a column with different letters are significantly different (*p* < 0.05).

**Table 2:** Total phenolic content (µg/g dry weight), and phytochemical content (µg/g dry weight) in the untreated (control) and HPP treated potato cultivars.

Potato Cultivar	Sample Type	Polyphenols						Glycoalkaoids	
		Total Phenolic Content	Ferulic Acid	Chlorogenic Acid	Caffeic acid	*p*-Coumaric Acid	Rutin	α-Chaconine	α-Solanine
Saxon	control	1098 ± 36 ^a^	67.39 ± 1.40 ^a^	106.72 ± 7.28 ^a^	1.23 ± 0.16 ^a^	23.79 ± 0.04 ^a^	0.31 ± 0.07 ^a^	1.05 ± 0.18 ^a^	1.48 ± 0.19 ^a^
	HPP-treated	1342 ± 35 ^b^	97.41 ± 1.26 ^b^	105.68 ± 9.29 ^a^	9.86 ± 0.05 ^b^	23.94 ± 0.21 ^a^	0.07 ± 0.01 ^b^	2.60 ± 0.42 ^a^	2.99 ± 0.24 ^b^
Gemson	control	1145 ± 24 ^a^	69.19 ± 1.34 ^a^	131.92 ± 4.00 ^b^	0.22 ± 0.06 ^c^	24.04 ± 0.29 ^a^	0.73 ± 0.09 ^c^	19.52 ± 1.15 ^b^	18.07 ± 1.77 ^c^
	HPP-treated	1417 ± 45 ^b^	64.67 ± 0.76 ^c^	120.30 ± 3.65 ^c^	5.29 ± 0.09 ^d^	24.69 ± 0.16 ^a^	1.14 ± 0.2 ^d^	22.29 ± 1.42 ^b^	21.36 ± 1.65 ^c^
Rooster	control	796 ± 18 ^c^	63.11 ± 1.48 ^c^	119.21 ± 13.82 ^a^	0.47 ± 0.13 ^e^	23.78 ± 0.08 ^a^	2.68 ± 0.18 ^e^	1.52 ± 0.25 ^c^	3.99 ± 0.68 ^d^
	HPP-treated	976 ± 6 ^d^	60.42 ± 0.41 ^d^	95.95 ± 5.08 ^d^	4.88 ± 0.34 ^d^	24.78 ± 0.57 ^a^	3.02 ± 0.39^f^	2.49 ± 0.76 ^a c^	3.86 ± 0.44 ^d^
Kerr’s Pink	control	830 ± 11 ^c^	56.20 ± 0.36 ^e^	139.53 ± 9.83 ^b^	0.04 ± 0.01 ^e^	23.65 ± 0.06 ^a^	1.79 ± 0.27^g^	7.36 ± 0.60 ^d^	3.28 ± 0.26 ^d^
	HPP-treated	984 ± 11 ^d^	59.87 ± 1.74^f^	88.73 ± 1.57 ^e^	2.66 ± 0.11^f^	24.22 ± 0.25 ^a^	0.91 ± 0.18 ^d^	8.06 ± 0.15 ^d^	3.48 ± 0.18 ^d^

Values are mean ± standard deviation. Mean values in a column with different letters are significantly different (*p* < 0.05).

## Supplementary Materials

The following are available online at https://www.mdpi.com/2304-8158/8/10/517/s1, Figure S1: HPLC-Q-Tof-MS profiling of the phytochemicals in the potato Saxon extract. Shown in the inset is the LC chromatogram where the polyphenols and glycoalkaloids elute between 2–9 min, Figure S2: UPLC-TQD-MS quantification of polyphenols in the different potato cultivars prior to (left column) and post-HPP (right column). As evident from the total ion chromatograms (TIC) that chlorogenic acid is the most abundant polyphenol in potatoes, which is reduced in the post-HPP samples with an increase in caffeic acid, Table S1: Tentative identification of phytochemicals from the potato (Saxon) extract using accurate mass measurement and MS/MS fragment ions.
