# Supplementary material for: The Effect of High Pressure Processing on Polyphenol Oxidase Activity, Phytochemicals and Proximate Composition of Irish Potato Cultivars
Source: Foods. 2019 Oct 19;8(10):517. doi: 10.3390/foods8100517 (PMC6835228; doi:10.3390/foods8100517)
Supplement: Supplementary file 1 [file foods-08-00517-s001.pdf]

foods

MDPI

Communication

The effect of high pressure processing on polyphenol oxidase activity, phytochemicals and proximate composition of Irish potato cultivars

Konstantina Tsikrika[1], Nora O'Brien[2], and Dilip K. Rai[1*]

[1]Department of Food Biosciences, Teagasc Food Research Centre, Ashtown, Dublin, D15 KN3K, Ireland; Konstantina.tsikrika@teagasc.ie

[2]School of Food & Nutritional Sciences, University College Cork, College Road, Cork T12 K8AF, Ireland; nob@ucc.ie

* Correspondence: dilip.rai@teagasc.ie ; Tel.: +353-1-8059500 (D.K.R)

Received: date; Accepted: date; Published: date

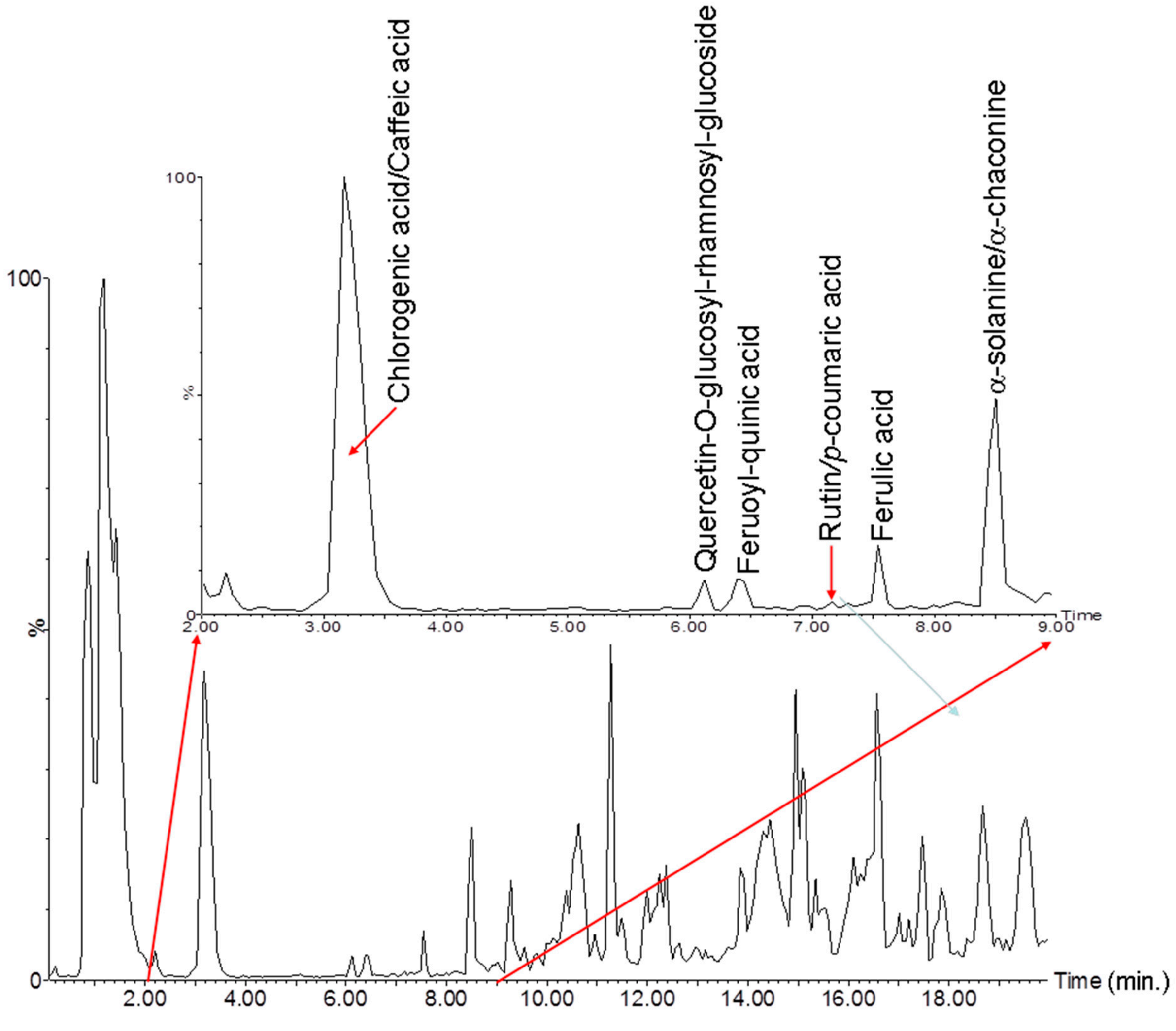

Figure S1: HPLC-Q-Tof-MS profiling of the phytochemicals in the potato (Saxon) extract. Shown in the inset is the LC chromatogram where the polyphenols and glycoalkaloids elute between 2-9 minutes.

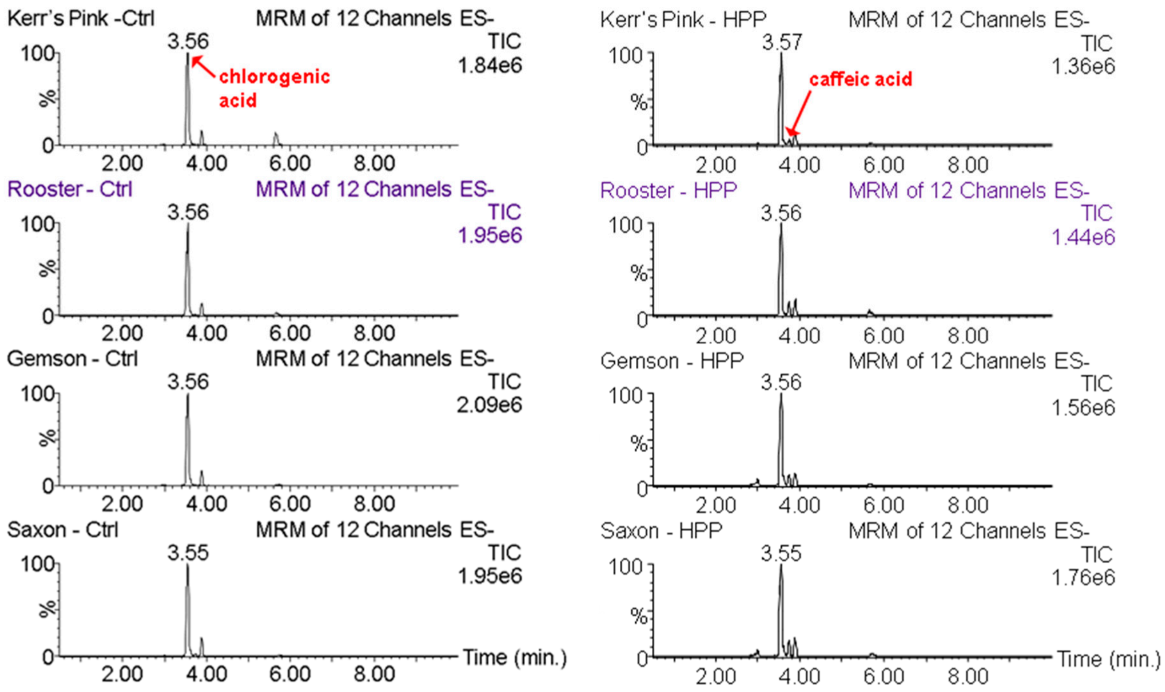

Figure S2: UPLC-TQD-MS quantification of polyphenols in the different potato cultivars prior to (left column) and post-HPP (right column). As evident from the total ion chromatograms (TIC) that chlorogenic acid is the most abundant polyphenol in potatoes, which is reduced in the post-HPP samples with an increase in caffeic acid.

Table S1. Tentative identification of phytochemicals from the potato (Saxon) extract using accurate mass measurement and MS/MS fragment ions

RT (min.)	Observed $[M-H]^-$ (*m/z*)	Calculated $[M-H]^-$ (*m/z*)	Molecular formula	MS/MS ions (*m/z*)	Tentative Identification
0.88	158.9764	158.9752	$C_5H_4O_4S$	130.99, 115.00	Dihydroxythiophene-carboxylic acid
0.98	191.0541	191.0556	$C_7H_{12}O_6$	147.05	Quinic acid
2.17	203.8050	203.0821	$C_{11}H_{12}N_2O_2$	159.09	Tryptophan
3.17	353.0858	353.0873	$C_{16}H_{18}O_9$	191.08, 173.06, 135.06	Chlorogenic acid
3.46	179.0341	179.0344	$C_9H_8O_4$	135.04	Caffeic acid
6.12	771.1978	771.1984	$C_{33}H_{40}O_{21}$	300.03	Quercetin-*O*-glucosyl-rhamnosyl-glucoside
6.39	367.1015	367.1029	$C_{17}H_{20}O_9$	193.04, 191.05, 173.05	Feruoyl-quinic acid
6.44	625.1384	625.1405	$C_{27}H_{30}O_{17}$	300.03, 191.05	Quercetin- *O*-di-glucoside
7.16	609.1442	609.1456	$C_{27}H_{30}O_{16}$	301.03	Rutin
7.19	163.0387	163.0395	$C_9H_8O_3$	119.05	*p*-coumaric acid
7.51	193.0488	193.0501	$C_{10}H_{10}O_4$	134.04, 117.04	Ferulic acid
8.42	866.4885	866.4902	$C_{45}H_{73}NO_{15}$	704.51, 701.32, 558.47	α-solanine
8.48	850.4915	850.4953	$C_{45}H_{73}NO_{14}$	704.41, 422.14	α–chaconine

© 2019 by the authors. Submitted for possible open access publication under the terms and conditions of the Creative Commons Attribution (CC BY) license (http://creativecommons.org/licenses/by/4.0/).
